# Angiogenic Effects of Secreted Factors from Periodontal Ligament Stem Cells

**DOI:** 10.3390/dj9010009

**Published:** 2021-01-15

**Authors:** Kengo Iwasaki, Keiko Akazawa, Mizuki Nagata, Motohiro Komaki, Yihao Peng, Makoto Umeda, Tetsuro Watabe, Ikuo Morita

**Affiliations:** 1 Institute of Dental Research, Osaka Dental University, Osaka 573-1121, Japan; 2 Department of Nanomedicine (DNP), Graduate School of Medical and Dental Sciences, Tokyo Medical and Dental University, Tokyo 113-8510, Japan; m.komaki@kdu.ac.jp; 3 Department of Periodontology, Graduate School of Medical and Dental Sciences, Tokyo Medical and Dental University (TMDU), Tokyo 113-8510, Japan; akakperi@tmd.ac.jp (K.A.); mizukin@umich.edu (M.N.); 4 Yokohama Clinic, Kanagawa Dental University, Yokohama Clinic, Kanagawa, Yokohama 221-0835, Japan; 5 Graduate School of Dentistry, Department of Periodontology, Osaka Dental University, Osaka 573-1121, Japan; pengyh910806@gmail.com; 6 Department of Periodontology, Osaka Dental University, Osaka 573-1121, Japan; umeda-m@cc.osaka-dent.ac.jp; 7 Department of Biochemistry, Graduate School of Medical and Dental Sciences, Tokyo Medical and Dental University (TMDU), Tokyo 113-8510, Japan; t-watabe@umin.ac.jp; 8 Ochanomizu University, Tokyo 112-8610, Japan; morita.ikuo@ocha.ac.jp

**Keywords:** periodontal disease, regeneration, stem cells, periodontal ligament, angiogenesis

## Abstract

Periodontal disease is a chronic inflammation of tooth-supporting tissues, and the destruction of these tissues results in tooth loss. Regeneration of periodontal tissues is the ultimate goal of periodontal treatment. We previously reported that transplantation of conditioned medium (CM) of periodontal ligament stem cells (PDLSCs) demonstrated the enhancement of periodontal tissue regeneration, compared to CM from fibroblasts (Fibroblast-CM). We hypothesized that the angiogenic effects of PDLSC-CM might participate in the enhanced wound healing of periodontal tissues. The aim of this study was to investigate the effect of PDLSC-CM on the functions of endothelial cells. PDLSCs were cultured from periodontal ligament tissues obtained from healthy volunteers. Human gingival epithelial cells, dermal fibroblasts, osteoblasts, and umbilical vein endothelial cells (HUVECs) were purchased from commercial sources. The functions of endothelial cells were examined using immunostaining of Ki67, observation of nuclear fragmentation and condensation (apoptosis), and network formation on Matrigel. Vascular endothelial cell growth factor (VEGF) level was measured using an ELISA kit. HUVECs demonstrated higher cell viability in PDLSC-CM when compared with those in Fibroblast-CM. HUVECs demonstrated a higher number of Ki67-positive cells and lower apoptosis cells in PDLSC-CM, compared to Fibroblast-CM. Additionally, HUVECs formed more capillary-like structures in PDLSC-CM than Fibroblast-CM. PDLSC-CM contained higher levels of angiogenic growth factor, VEGF, than Fibroblast-CM. Our results showed that PDLSC-CM increased cell viability, proliferation, and capillary formation of HUVECs compared to Fibroblast-CM, suggesting the angiogenic effects of PDLSC-CM, and the effect is a potential regenerative mechanism of periodontal tissues by PDLSC-CM.

## 1. Introduction

Periodontal disease is a chronic inflammatory disease of tooth-supporting tissues, primarily caused by periodontopathic bacterial infection [1,2]. In the progressive form of the disease, extensive loss of tooth-supporting tissues can be found, and diseased teeth are extracted due to their considerable mobility [3]. In addition to the negative effects on masticatory function, recent studies have suggested that the chronic inflammation of periodontal tissues may contribute toward several systemic diseases, including diabetes and cardiovascular disease, and have emphasized the importance of periodontal treatment in terms of systemic health [4,5].

Because periodontal disease demonstrates the destruction of periodontal tissues, regeneration of the lost tissues is the ultimate treatment goal of periodontal therapy. Therefore, many regenerative strategies have been examined for their regenerative potential, and some have been applied clinically, including guided tissue regeneration (GTR) and enamel matrix derivatives [6,7]. These regenerative treatments have succeeded in the regeneration of periodontal tissues; however, the amount of periodontal tissues that is regenerated and an indication of these regenerative methods are limited [8,9]. Therefore, there is an extensive need for new periodontal regenerative treatments.

Based on the progression of stem cell (SC) research and tissue engineering technologies, the possibility of tissue regeneration using SC, expanded ex vivo, has been proposed for various pathological settings. Mesenchymal SCs (MSCs) were originally isolated from bone marrow as colony-forming and plastic-adherent fibroblastic cell populations [10]. Because MSCs exhibit differentiation into multiple mesenchymal lineages, such as osteoblasts, adipocytes, and chondrocytes, in vitro, they are widely accepted as putative adult SCs of mesenchymal tissues [11,12]. Seo et al. (2004) isolated cells with MSC-like properties from the periodontal ligament (PDL) and named them periodontal ligament stem cells (PDLSCs) [13]. They also demonstrated that transplanted PDLSCs formed new cementum-PDL-like tissues in vivo and suggested that PDLSCs are adult SCs in PDL. Since this discovery, increasing attention has been paid to PDLSCs as a candidate cell type for the cell-based treatment of periodontal disease. Many studies have demonstrated that PDLSCs can differentiate into periodontal tissue-forming cells, such as cementoblasts, osteoblasts, and fibroblasts, and animal studies have demonstrated successful periodontal tissue regeneration after the transplantation of the SCs into periodontal bony defects, and human clinical studies have already begun [14,15,16,17,18]. We previously reported that the transplantation of PDLSCs induced the regeneration of periodontal tissues in rat periodontal defect models [19,20]. In these studies, we also found that the engraftment of PDLSCs in periodontal defects after transplantation was limited despite periodontal regeneration being observed, suggesting the limited contribution of direct cell differentiation to periodontal regeneration [20]. Therefore, we examined the involvement of paracrine factors from PDLSCs in periodontal tissue regeneration and found that transplantation of the conditioned medium (CM) obtained from PDLSCs enhanced periodontal tissue regeneration [21]. Additionally, the levels of regenerated alveolar bone were higher in PDLSC-CM-transplanted defects compared to in CM from dermal fibroblasts (Fibroblast-CM), although regeneration mechanisms underlying CM transplantation were unclear.

Based on the above background, to clarify the regenerative mechanism of PDLSC-CM, we compared the effect of PDLSC-CM and Fibroblast-CM on various cell types, which may participate in wound healing of periodontal tissues, including gingival epithelial cells, endothelial cells, and osteoblasts. As a result, we found that PDLSC-CM significantly enhanced the viability in endothelial cells, which was more than that observed in epithelial cells and osteoblasts. Then, we focused on endothelial cells and examined the angiogenic effects of PDLSC-CM, comparing with those of Fibroblast-CM in this study.

## 2. Materials and Methods

### 2.1. Cell Culture and CM Collection

Considering the variations of the effects of PDLSC-CM among donor’s teeth, we used PDLSC-CMs taken from three PDLSC lines for each experiment in this study [22,23,24]. In order to establish PDLSC lines, PDL tissue was collected from 8 extracted premolars of healthy volunteers (14–28 years old, average 18 years old, 1 male, and 7 females). It was confirmed that all these premolars were free from any symptoms of periodontal disease. This study protocol was approved by the ethical committee for clinical research at Tokyo Medical and Dental University (TMDU) (#723) on 10 July 2013. All experiments were performed in accordance with the Ethical Guidelines for Medical and Health Research Involving Human Subjects by the Japanese Ministry of Health, Labour, and Welfare. PDLSCs were cultured from PDL tissues using the collagenase/dispase digestion method [13,25]. Cells were expanded in alpha minimal essential medium (αMEM) (Life Technologies, Carlsbad, CA, USA) with 15% fetal bovine serum (FBS), GlutaMAX (Life Technologies), and antibiotic-antimycotic solution (Life Technologies). To minimize the influence of inflammation of PDL and aging of the donor on data, we isolated PDLSCs from healthy PDL tissues from young donors, and cells with a passage number of less than four were used in this study. Multiple differentiation capacity of PDLSCs was confirmed prior to the experiments. For the induction of osteoblastic differentiation, the culture medium was changed to αMEM containing L-ascorbic acid (L-ascorbic acid 2-phosphate) (50 μg/mL) (Sigma, St Louis, MO, USA), dexamethasone (10^−8^ M) (Sigma), and β-glycerophosphate (10 mM) (Sigma) for 21 days. Calcium deposit was stained using Von Kossa staining kit (Polysciences, Warrington, PA, USA). For the induction of adipocyte differentiation, the adipocyte differentiation medium was used, according to instructions from the manufacturer (Lonza, Walkersville, MD, USA). After the induction, cells were fixed with 10% formalin solution (Sigma) and washed with phosphate-buffered saline (PBS) twice. Then, Oil Red O solution (Sigma) was added for 30 min, followed by rinsing plate with PBS. Photographs of culture plates were taken with Keyence BZ800 (Keyence, Osaka, Japan). Human gingival epithelial cells (HGE) (CELLnTEC, Berne, Switzerland), dermal fibroblasts (Lonza), osteoblasts (HOB) (PromoCell, Heidelberg, Germany), and umbilical vein endothelial cells (HUVECs, CELLnTEC) were purchased and used in this study. HUVECs expressing green fluorescent protein (HUVEC-GFP) were established and cultured, as previously described [26]. For the collection of CM, the culture medium was changed to low-glucose Dulbecco’s modified Eagle’s medium (DMEM, Life Technologies) at 70–80% confluence. After 48 h of incubation, culture supernatants were collected, and cells and debris were removed by centrifugation and filtration. CM was concentrated using ultrafiltration with a 10 kDa cut-off value (Millipore, Billerica, MA, USA).

### 2.2. Cell Viability

Cells were seeded in 96-well plates (2500 to 5000 cells/well) for 18 h, and the culture medium was changed to each CM. Cell viability was examined 48 h after incubation with various conditioned media using a Cell Counting Kit (Dojindo, Kumamoto, Japan).

### 2.3. Ki67 Immunostaining and DAPI Staining

Cells were fixed in 4% paraformaldehyde in phosphate buffer and permeabilized in 0.25% Triton-X in PBS. Goat serum-PBS (10%) was used for blocking, and mouse anti-Ki67 antibody (Dako, Carpinteria, CA, USA) and goat anti-mouse IgG Alexa 488 (Santa Cruz Biotechnology, Santa Cruz, CA, USA) were used as the primary and secondary antibodies, respectively. Cells were observed under the fluorescence microscope Keyence BZ800 (Keyence, Osaka, Japan) after nuclear staining with 4′,6-Diamidino-2-phenylindole dihydrochloride (DAPI). Apoptosis was determined by the observation of nuclear condensation after DAPI staining [27]. The ratio of Ki67-positive cells and cells with nuclear alterations was calculated from the total nuclear counts and number of Ki67-positive and nuclear alteration cells.

### 2.4. Network Formation

First, 100 μL of growth factor-reduced Matrigel (BD Bioscience, San Jose, CA, USA) was added to a 48-well plate and solidified at 37 °C. After solidification, 2 × 10^4^ HUVEC-GFP cells were seeded in 125 μL of endothelial cell growth medium (EGM)-2. Three hours later, the medium was changed to various CMs. Network formation was observed under a fluorescence microscope Keyence BZ800 (Keyence). The number of meshes was counted, as previously described [28].

### 2.5. Measurement of Growth Factors

Taking advantage of higher sensitivity in protein measurement of liquid samples, we used the ELISA method for the detection of growth factors. The amount of vascular endothelial growth factor (VEGF) in conditioned media was measured using an ELISA kit (R&D Systems, Minneapolis, MN, USA).

### 2.6. Statistical Analysis

Data are expressed as mean ± S.D. All statistical analyses were performed using StatView software (SAS Institute, Middleton, MA, USA). Analysis of variance (ANOVA) was first determined among the experimental groups. When ANOVA indicated significance, Fisher’s least significant difference (LSD) or Tukey’s/Kramer test was then conducted. For the comparison of the two groups, Student’s *t*-test was used.

## 3. Results

### 3.1. Isolation and Characterization of PDLSCs

**Figure 1:**
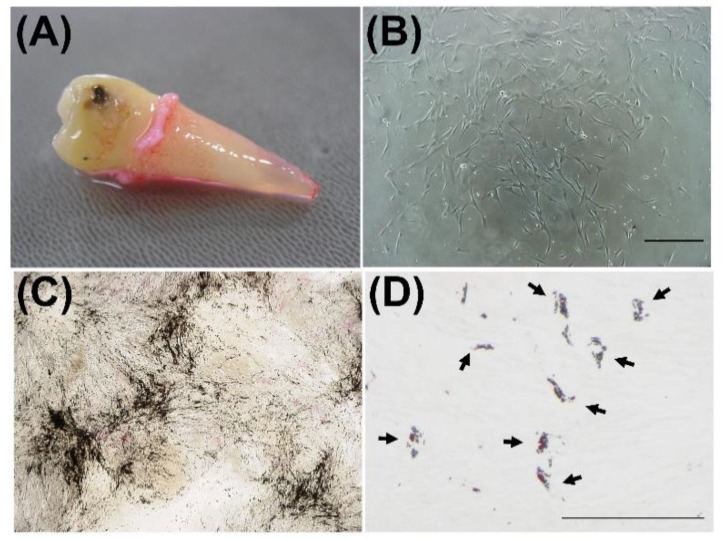
Isolation and characterization of periodontal ligament stem cells (PDLSCs). PDLSCs were isolated from the periodontal ligament (PDL) and characterized. PDL tissues were obtained from the extracted human tooth (**A**). Colony-forming fibroblastic cells were observed 9 days after enzymatic digestion of PDL (**B**). Images after von Kossa staining and Oil Red O staining of PDLSC culture are shown (**C**,**D**). Three weeks after the induction of osteoblastic differentiation, PDLSCs demonstrated von Kossa-positive calcium deposit formation. Four weeks after the induction of adipocyte differentiation, PDLSCs demonstrated Oil Red O-positive lipid accumulation in cytosolic space. Black arrows: Oil Red O-positive lipid droplets, Bar = 300 μm (**B**) and 100 μm (**C**,**D**).

We isolated PDLSCs from the healthy extracted tooth (Figure 1A). After seeding of single-cell suspension of PDL-derived cells on the plastic dish, we observed colony-forming fibroblastic cells one to two weeks later (Figure 1B). These colony-forming cells demonstrated prominent calcium deposits, which were positive for von Kossa staining after in vitro induction of osteoblastic differentiation (Figure 1C). Additionally, these cells also exhibited Oil Red O-positive lipid accumulation in cytosolic space after the induction of adipocyte differentiation, as shown in Figure 1D. We used these colony-forming PDL cells with multiple differentiation capacity as PDLSCs.

### 3.2. Effect of CMs on the Cell Viability of Periodontal Tissue-Composing Cells

**Figure 2:**
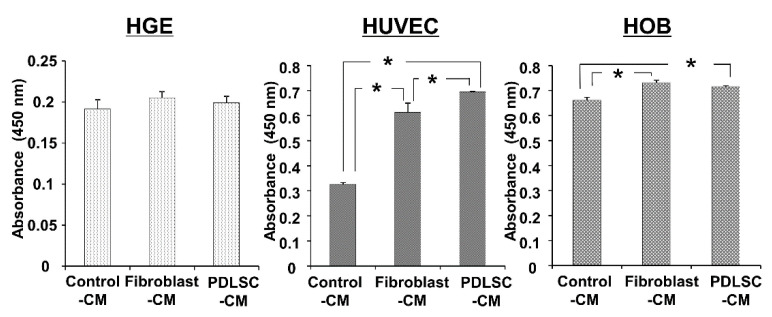
Effect of CM on the cell viability of HGEs, HUVECs, and HOBs. HGEs, HUVECs, and HOBs were incubated in Control-CM, Fibroblast-CM, and PDLSC-CM for 48 h, and cell viability was examined using water soluble tetrazolium salt (WST)-8. Cell viability is shown as the absorbance value at 450 nm. PDLSC-CM and Fibroblast-CM increased cell viability of HUVEC and HOB. HUVEC exhibited higher viability when cultured in PDLSC-CM compared to in Fibroblast-CM. (* *p* < 0.05, HGE = human gingival epithelial cells, HUVECs = human umbilical cord vein endothelial cells, HOB = human osteoblasts, CM = conditioned medium, PDLSCs = periodontal ligament stem cells). All experiments were performed in triplicates, and the representative data from an independent experiment performed three times were shown. (*n* = 3).

To compare the effect of CMs from PDLSCs and fibroblasts on various cell types, which may participate in the wound healing of periodontal tissues, we examined the cell viability of gingival epithelial cells (HGE), endothelial cells (HUVEC), and osteoblast (HOB) in PDLSC-CM and Fibroblast-CM. As demonstrated in Figure 2, both CMs did not influence the viability of gingival epithelial cells. On the other hand, the viability of endothelial cells and osteoblasts was increased in both PDLSC-CM and Fibroblast-CM. When we compared PDLSC-CM and Fibroblasts-CM, PDLSC-CM significantly enhanced the cell viability of HUVECs than Fibroblast-CM. Thus, we focused on the effect of CMs on endothelial cells in the following experiments.

### 3.3. Effect of CM on the Apoptosis of HUVECs

**Figure 3:**
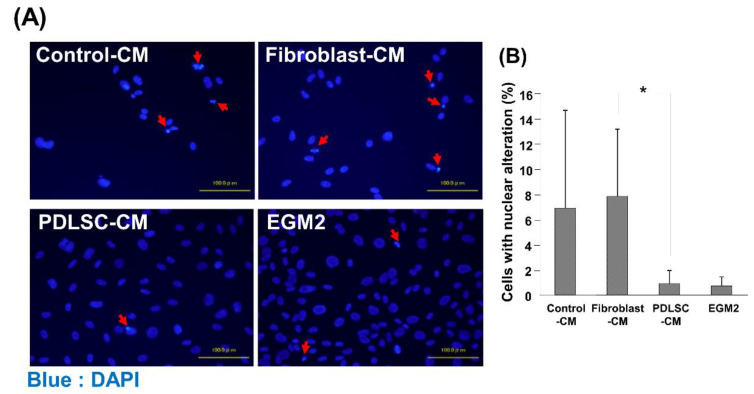
Cell death of HUVECs cultured in various CMs. Representative images of HUVECs with alterations in nuclear morphology after DAPI staining are shown, including nuclei fragmentation and condensation (**A**). The number of HUVECs with nuclear alteration was quantified (**B**). A lower number of cells with alterations of nuclear morphology was identified in HUVECs cultured with PDLSC-CM, compared to Control-CM and Fibroblast-CM. Red arrows: alterations of nuclear morphology (* *p* < 0.05) (HUVECs = human umbilical cord vein endothelial cells, DAPI = 4′,6-Diamidino-2-phenylindole dihydrochloride, CM = conditioned medium, PDLSCs = periodontal ligament stem cells, EGM2 = endothelial cell growth medium 2). Data from an independent experiment performed three times were shown. (*n* = 3).

It has previously been demonstrated that MSCs have anti-apoptotic functions [29,30]. Therefore, as a potential factor causing changes in the viability of HUVECs, the effects of various CMs on apoptosis of HUVECs were examined. HUVECs demonstrated a lower number of cells with nuclear condensation in PDLSC-CM compared to in Control-CM and Fibroblast-CM (Figure 3). The percentage of nuclear alterations in the PDLSC-CM group was as low as that of EGM-2, the growth medium of endothelial cells, which served as a positive control.

### 3.4. Effect of CM on the Cell Proliferation of HUVECs

**Figure 4:**
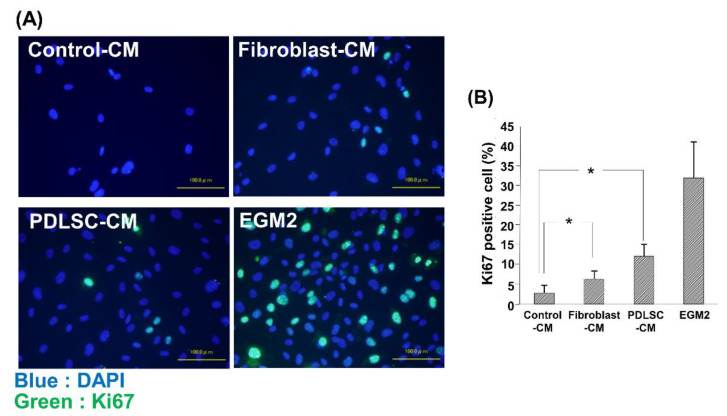
Ki67 staining of HUVECs cultured in various CMs. Representative merged images of Ki67 and nucleus (DAPI) staining in HUVECs cultured in Control-CM, Fibroblast-CM, PDLSC-CM, and EGM2 (**A**). The number of proliferating HUVECs was examined using Ki67 immunostaining (**B**). Quantification of the ratio of Ki67-positive cells. An increased number of Ki67-positive cells was observed in HUVECs cultured with PDLSC-CM compared to Control-CM. HUVECs cultured in EGM2 served as positive control. (* *p* < 0.05) (HUVECs = human umbilical cord vein endothelial cells, DAPI = 4′,6-Diamidino-2-phenylindole dihydrochloride, CM = conditioned medium, PDLSC = periodontal ligament stem cells, EGM2 = endothelial cell growth medium 2). Data from an independent experiment performed three times were shown. (*n* = 3).

Next, we examined the effects of various CMs on the proliferation of HUVECs by examining the number of positive cells for nuclear antigen Ki67. As demonstrated in Figure 4, we observed a higher number of Ki67-positive cells in Fibroblast-CM than in Control-CM. The Ki67-positive cell number was further increased in PDLSC-CM compared to in Fibroblast-CM, suggesting enhancement of the proliferation of HUVECs by PDLSC-CM.

### 3.5. Effect of CM on the Network Formation of HUVECs

**Figure 5:**
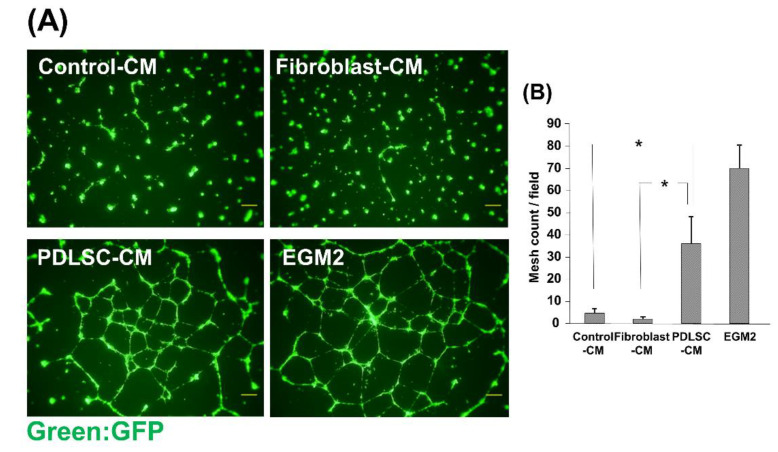
Effect of CM on the network formation of HUVECs. Fluorescence microscopic images of HUVEC networks (**A**). HUVECs-GFP were cultured on Matrigel in the presence of Control-CM, Fibroblast-CM, PDLSC-CM, and EGM2. The number of mesh structures formed by HUVECs was quantified (**B**). HUVECs in PDLSC-CM formed more capillary-like networks than those in Control-CM and Fibroblast-CM. (* *p* < 0.05) (HUVECs = human umbilical cord vein endothelial cells, CM = conditioned medium, PDLSCs = periodontal ligament stem cells, EGM2 = endothelial cell growth medium 2). Data from an independent experiment performed three times were shown. (*n* = 3).

To investigate the effects of CM on the angiogenic function of HUVECs, we examined the capillary-like network formation of HUVECs on Matrigel in the presence of various CMs. HUVEC-GFP formed capillary-like networks on Matrigel in the positive control EGM2 medium, and many mesh-like structures were observed (Figure 5). Mesh-like structures were hardly observed in Control-CM and Fibroblast-CM. On the other hand, it was found that HUVECs formed multiple mesh structures and that capillary-like structure formation was augmented in PDLSC-CM. These results suggest the angiogenic effects of PDLSC-CM on HUVECs.

### 3.6. Angiogenic Factor in PDLSC-CM

**Figure 6:**
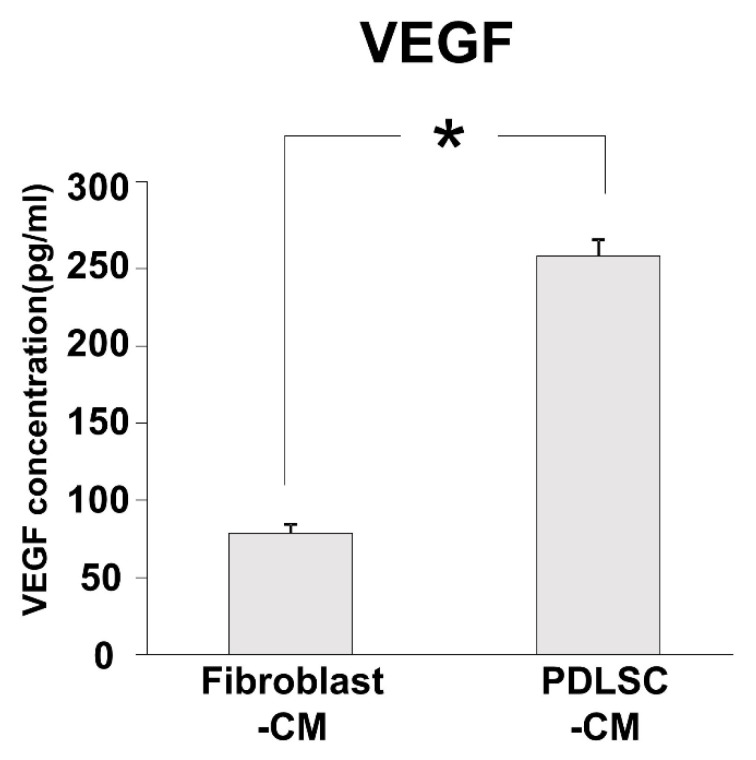
The concentration of VEGF in CM. Concentrations of the angiogenesis-related growth factor, VEGF, in Fibroblast-CM and PDLSC-CM. A greater amount of VEGF was found in PDLSC-CM than in Fibroblast-CM. (* *p* < 0.05) (CM = conditioned medium, PDLSCs = periodontal ligament stem cells, VEGF = vascular endothelial growth factor). Experiments were performed in triplicates, and data from an independent experiment performed three times were shown. (*n* = 3).

Our results demonstrated that viability, proliferation, and capillary-like structure formation were all enhanced in PDLSC-CM compared to in Fibroblast-CM. Next, we examined the amounts of important angiogenic factor, VEGF, in PDLSC-CM and Fibroblast-CM. The protein level of VEGF was higher in PDLSC-CM than in Fibroblast-CM, as demonstrated in Figure 6.

## 4. Discussion

In the present study, we found that HUVECs demonstrated lower apoptosis, higher viability, a Ki67-positive cell number, and capillary formation in PDLSC-CM, compared to in Fibroblast-CM. Additionally, an important angiogenic factor, VEGF, was detected at higher levels in PDLSC-CM than in Fibroblast-CM. These results strongly suggest that PDLSC-CM enhances angiogenesis by modulating the behavior of endothelial cells.

The EGM2, which was used as a positive control medium in this study, contained various growth factors, vitamins, and chemicals, such as bFGF, VEGF, ascorbic acid, and heparin, and was optimized for the various functionalities of endothelial cells in vitro. Our results demonstrated that in the EGM2 medium, HUVECs exhibited lower apoptosis and a higher number of Ki67-positive cells and network formation. We did not add exogenous factors into DMEM-based CM, including Control-CM, Fibroblast-CM, and PDLSC-CM. Therefore, HUVECs showed relatively higher cell death and lower cell proliferation and network formation in these CMs. However, among these CMs, PDLSC-CM demonstrated different trends of effects on HUVECs compared with those of Control-CM and Fibroblast-CM. This tendency was observed in the number of apoptotic cells and network formation, where PDLSC-CM and EGM-2 showed lower apoptosis and higher network formation, and Control-CM and Fibroblast-CM demonstrated opposite results. These results strongly indicate the angiogenic effects of PDLSC-CM.

PDLSCs are colony-forming, plastic adherent fibroblastic cells and have similar characteristics to bone marrow-derived MSCs, such as differentiation potential into osteoblasts, adipocytes, and chondrocytes in vitro and cell surface antigen expression profiles [13,29,31,32]. Additionally, it was demonstrated that MSCs release various angiogenic factors. In a previous study, we demonstrated that PDLSC-CM contained a number of important proteins in angiogenesis using protein array analysis [21]. In this study, we found a higher level of VEGF in PDLSC-CM than in Fibroblast-CM. VEGF is one of the most important angiogenic growth factors, which directly induces the growth and migration of endothelial cells [33,34]. The higher level of VEGF in PDLSC-CM may explain the enhancement of cell viability, Ki67-positive cell number, and network formation of HUVECs in PDLSC-CM compared to Fibroblast-CM.

Several previous studies have reported the angiogenic effects of PDLSCs. Bae et al. (2017) demonstrated that PDLSCs enhanced blood vessel formation by the transplantation of HUVECs in vivo, suggesting the supportive effect of PDLSCs in angiogenesis [35]. Additionally, Yeasmin et al. (2014) found that PDLSCs abundantly produced VEGF compared to SCs from human exfoliated deciduous teeth and bone marrow MSCs [36]. These findings suggest the angiogenic function of PDLSCs and are in agreement with our findings that PDLSC-CM enhances the proliferation and angiogenic potential of HUVECs.

We previously reported that the transplantation of PDLSC-CM into periodontal defects resulted in enhanced periodontal tissue regeneration compared to Fibroblast-CM transplantation [21]. As PDLSC-CM contains various angiogenic factors, it is assumed that angiogenic factors in PDLSC-CM may explain the enhanced regenerative process of periodontal tissues compared to Fibroblast-CM. Angiogenesis is an important step in wound-healing, and it has been postulated that the enhancement of angiogenesis may be favorable via the supplementation of oxygen and nutrients to healing tissues. Our findings showing higher cell viability, a Ki67-positive cell number, and network formation in PDLSC-CM suggest the higher angiogenic effect of PDLSC-CM, and the effect is a potential regenerative mechanism of enhanced periodontal tissue formation by PDLSC-CM transplantation compared to Fibroblast-CM. Additionally, now the clinical trials for periodontal regeneration by PDLSC transplantation are underway, and this study provides information regarding the regenerative mechanism by PDLSC transplantation.

## 5. Conclusions

In this study, we found that HUVECs exhibited higher viability, a Ki-67-positive cell number, network formation, and lower apoptotic cell death in PDLSC-CM than in Fibroblast-CM, and PDLSC-CM contained an elevated level of VEGF compared to Fibroblast-CM. In conclusion, PDLSC-CM has a higher angiogenic effect on endothelial cells than Fibroblast-CM, which may explain the enhanced periodontal tissue regeneration with the transplantation of PDLSC-CM.

## Data Availability

The data that support the findings of this study are available from the corresponding author (K.I.) on reasonable request. The data are not publicly available due to ethical restrictions.
